# Association Between Dietary Inflammatory Index and Sex Hormone Binding Globulin and Sex Hormone in U.S. Adult Females

**DOI:** 10.3389/fpubh.2022.802945

**Published:** 2022-04-15

**Authors:** Nuozhou Liu, Ying Feng, Xinyao Luo, Xue Ma, Fang Ma

**Affiliations:** ^1^West China School of Medicine, West China Hospital, Sichuan University, Chengdu, China; ^2^West China School of Basic Medical Sciences and Forensic Medicine, Sichuan University, Chengdu, China; ^3^Department of Pediatric Urology, West China Hospital, Sichuan University, Chengdu, China; ^4^Key Laboratory of Birth Defects and Related Diseases of Women and Children (Sichuan University), Ministry of Education, Center for Translational Medicine, West China Second University Hospital, Sichuan University, Chengdu, China; ^5^Department of Obstetrics and Gynecology, West China Second University Hospital, Sichuan University, Chengdu, China

**Keywords:** dietary inflammatory index, SHBG sex hormone-binding globulin, inflammation, diet, sex hormone, NHANES

## Abstract

**Context:**

It is still unknown whether the dietary inflammatory index (DII) is associated with sex hormones and sex hormone binding globulin (SHBG) in adult women.

**Objective:**

This study examined the association between DII and sex hormones and SHBG in U.S. adult women.

**Design and Participants:**

This was a cross-sectional study. A total of 2,092 female participants (age ≥ 20) from the 2013–2016 National Health and Nutrition Examination Survey were enrolled. Dietary inflammatory potential was assessed by DII based on 24-h dietary recall. SHBG was assessed using immuno-antibodies and chemo-luminescence, whereas sex hormones were measured by ID-LC–MS/MS.

**Results:**

The average DII was 0.21 ± 1.68, ranging from −4.54 (most anti-inflammatory) to 4.28 (most pro-inflammatory). After adjusting all covariates, a per-unit DII increase in DII tertile 3 was related to an 8.05 nmol/L SHBG decrease compared to DII tertile 1 (*P* = 0.0366). Subgroup analysis stratified by perimenopausal period found that this negative association remained strong but only existed in women before (β = −3.71, 95% CI: −7.43, −0.12, *P* = 0.0423) the perimenopausal period. Interaction terms were added to both subgroup analyses and found no significant heterogeneity among different body mass index (BMI) or perimenopausal groups (*P* > 0.05). Treshold analyses showed that the association of age with SHBG was an inverted *U*-shaped curve (inflection point: age = 50 yrs).

**Conclusion:**

A proinflammatory diet caused decreased SHBG. However, more well-designed studies are still needed to validate and verify the causal relationship between DII and sex hormones and SHBG.

## Introduction

Sex hormone-binding globulin (SHBG) is a 373-amino-acid glycoprotein primarily produced and secreted by the liver into the bloodstream, where it binds sex steroids and regulates their bioavailability. SHBG has also been confirmed to originate from the human central nervous system (1). Sex steroids in women are mainly produced by theca interna, granulosa lutein, and theca lutein cells in the ovary (2). Sex steroid disorder not only affects sexual characteristics but is also associated with hypertension and Alzheimer's disease (3, 4). A cross-sectional study estimated that U.S. national prevalence of low SHBG was 2.7% in women, which was linked with body mass index (BMI), excessive liver fat, diabetes, ethnicity, chronic obstructive pulmonary disease, coronary heart disease, and smoking (5, 6). Previous studies revealed that a low serum SHBG concentration was positively correlated with a higher risk of polycystic ovary syndrome (PCOS) and PCOS-related metabolic disturbances (7), breast cancer incidence and mortality (8), type 2 diabetes (T2D) (9), and cardiovascular disease risk (10). The decline in serum SHBG was associated with inflammation, since proinflammatory cytokines such as TNF-α and IL-1β could inhibit SHBG synthesis (11). Philip P Cavicchia et al. first proposed the dietary inflammatory index (DII) as a literature-derived dietary tool to measure individual dietary inflammatory potential (12). A higher DII score indicates a more proinflammatory diet with increased serum interleukin-6 (IL-6) and C-reactive protein (CRP), and a lower DII score predicts a more anti-inflammatory diet (13, 14). Accumulative studies have revealed that a higher DII is associated with cardiovascular disease, metabolic syndrome, and prostate and colorectal cancer (15–17).

There have been a large number of studies on the relationship between inflammation and sex hormones and SHBG. A retrospective cross-sectional study found that postmenopausal women's SHBG level was negatively associated with a proinflammatory state, while estradiol was inversely associated with a proinflammatory state (18). Female patients with chronic inflammatory diseases, such as diabetes, presented low plasma SHBG levels (19). SHBG could protect against metabolic syndrome by suppressing inflammation and lipid accumulation in macrophages and adipocytes (20). Regulatory T (Treg) cells inhibiting visceral adipose tissue (VAT) inflammation in mice were found to be sex specific and dependent on sex hormones (21). Estradiol could have an anti-inflammatory impact on the induced colitis mouse model *via* the estrogen receptor β signaling pathway (22).

Perimenopause in women could also change reproductive profiles, including sex hormones, SHBG, and sexual ability (23). Perimenopause is a time of transition for women at midlife, resulting from reduced secretion of the ovarian hormones estrogen and progesterone due to depletion of ovarian follicles. The Stages of Reproductive Aging Workshop (STRAW) defined perimenopause as variable menstrual cycles (>7 days different from normal) and high FSH values due to negative feedback (24). Sex steroids in this period were in fact highly variable (23). For instance, estradiol concentrations remained relatively stable in the early stages of perimenopause and fluctuated in the late stage. When perimenopause was finished, a dramatic decline in estradiol was observed. Women in this period might present many symptoms, including vaginal and sexual changes, hot flashes, changing mood and sleep quality, sex hormone disorder, and changed bleeding patterns (25, 26). A cohort study based on women in Massachusetts revealed that the median age at the perimenopausal period was 47.5 years, while the median age at the postmenopausal period was 51.3 years (27). However, the exact diagnosis of the perimenopausal period still relies critically on clinicians' judgments about menstrual history and age due to inaccuracies in a laboratory test (25).

Previous studies identified a negative association between DII and total testosterone in male adolescents and adults (28, 29), but there was no study considering the association between DII and sex hormones and SHBG in women. We aimed to identify this association among women based on data obtained from the National Health and Nutrition Examination Survey (NHANES), providing new insights into sex hormone and SHBG management from a clinical-nutritional perspective.

## Materials and Methods

### Study Design and Population

This study is a cross-sectional study based on NHANES, a nationwide and ongoing cross-sectional survey managed by the National Center for Health Statistics at the U.S. Centers for Disease Control and Prevention. NHANES was used to record the nutritional and health status of the U.S. population and conducted on a repeated 2-year cycle with appropriately settled sample weights. The NHANES research protocol was approved by the institutional review board and included the written informed consent of all participants, following the principles of the Declaration of Helsinki. In each 2-year cycle, data were categorized into five sections, including Demographics, Dietary, Examination, Laboratory, and Questionnaire. All data in our study are publicly available at https://www.cdc.gov/nchs/nhanes/.

**Figure 1:**
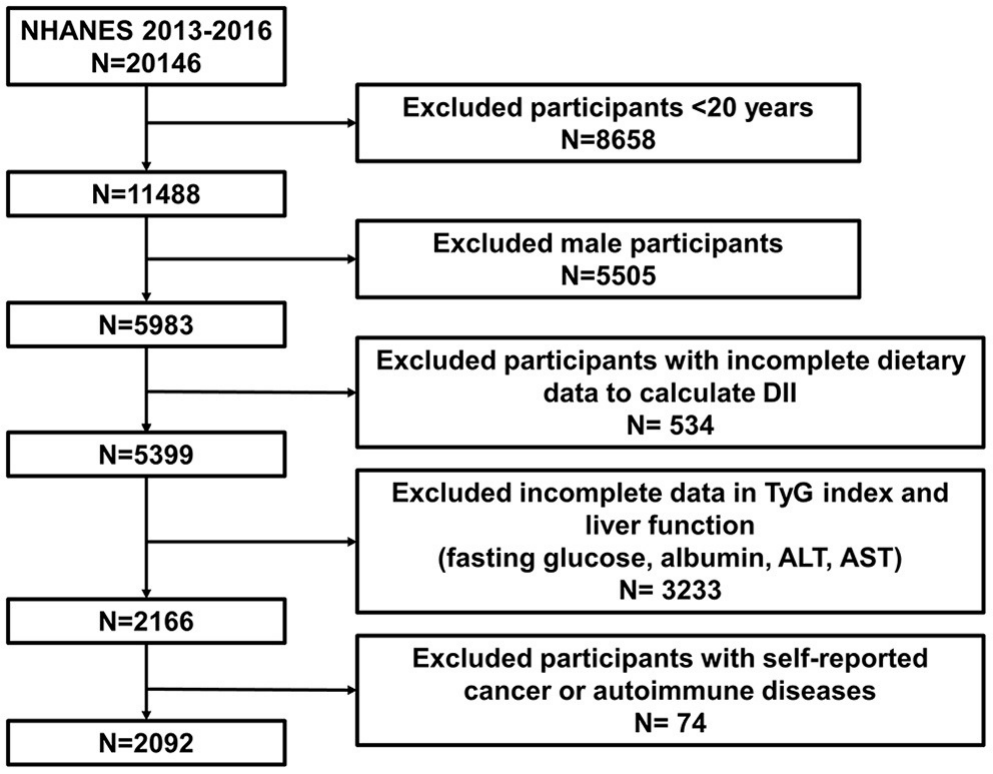
Flow diagram of sample selection from NHANES 2015–2016.

We obtained data from NHANES 2013–2016, which included enough information about sex hormones & SHBG and DII calculations. All females (age ≥ 20) who completed 24-h dietary recall and sex hormone and SHBG tests were enrolled in our study. If there was any absence of the information above, participants were excluded from our cohort. The detailed selection and exclusion procedure was as follows: (1) excluding male participants. (2) excluding female participants aged <20 years. (3) Pregnant participants or participants who were taking sex hormone medication were excluded. (4) Participants diagnosed with common tumors in women, including lung cancer, ovarian cancer, breast cancer, cervical cancer, and colon cancer, were excluded. (5) Participants with autoimmune diseases, including Hashimoto's thyroiditis and rheumatoid arthritis, were excluded. This procedure is also presented in Figure 1.

### Exposure and Outcome Definitions

**The** DII calculation was based on 24-h recall dietary data from NHANES, validated by the Nutrition Methodology Working Group (30). These dietary data contained information about food and drinks consumed during the 24-h before the interview. We calculated DII by following protocols presented by Shivappa et al. (31). A lower DII score indicated that the diet has more anti-inflammatory effects, while a higher DII score indicated a more proinflammatory diet (31). Previous studies revealed that using fewer than 30 food parameters for DII calculation would not affect DII's predictive capacity (28, 29, 32). For our study, 28 out of 45 food parameters available in NHANES were used to calculate DII, including alcohol, caffeine, protein, fiber, β-carotene, cholesterol, carbohydrates, energy, fats, n-3 fatty acid, n-6 fatty acid, poly-unsaturated fatty acid, mono-unsaturated fatty acid, saturated fat, thiamin, magnesium, zinc, selenium, iron, riboflavin, folic acid, vitamin A, vitamin B-6, vitamin B-12, vitamin C, vitamin D, vitamin E, and niacin. The DII was treated as a continuous variable in our study and stratified into three continuous DII tertiles for further statistical analysis. Serum SHBG measurement was based on the reaction of SHBG with immuno-antibodies and chemo-luminescence measurements of the reaction products that occur after two incubation periods and subjecting to a magnetic field. Then, the microparticles were captured on the electrode, where a chemiluminescence reaction occurred and could be measured by a photomultiplier tube. The readings were compared to an instrument- and lot-specific calibration curve. Total testosterone and estradiol measurements were conducted *via* isotope dilution liquid chromatography tandem mass spectrometry (ID-LC–MS/MS) for routine quantitation of serum total testosterone and estradiol, which was based on the National Institute for Standards and Technology's (NIST) reference method. Bioavailable testosterone calculation was based on Vermeulen's formula using serum albumin, SHBG, and total testosterone (33). The detailed measurement protocols are available at www.cdc.gov/nchs/nhanes/.

### Covariates

Our study employed age, race, energy and protein intake, smoking status, the ratio of family income to poverty (RIP), body mass index (BMI), education level, serum albumin, total bilirubin, white blood cell count, alanine aminotransferase (ALT), aspartate aminotransferase (AST), triglyceride-glucose (TyG) index, and marital status as covariates. Since SHBG is primarily synthesized and secreted by the liver, we used serum albumin, ALT, AST, and total bilirubin to represent liver function (34). According to previous studies (28), participants were categorized into 3 groups according to BMI, consisting of normal (BMI < 25 kg/m^2^), overweight (25 ≤ BMI ≤ 30 kg/m^2^) and obese (BMI > 30 kg/m^2^). We defined smoking status as never, former, and current *via* “Smoking-Cigarette Use” from questionnaire data in NHANES following Alharthi et al. (35). We further incorporated the TyG index to reflect the insulin resistance condition in each individual, which was closely related to SHBG (36, 37). The TyG index was quantified as Ln[triglycerides (mg/dl) ^*^ fasting glucose (mg/dl)/2], and a higher TyG index indicated a more serious insulin resistance condition (38). The perimenopausal period was defined as the period from age 45–55 in our study based on previous studies (25, 27, 39–41). Then, participants were similarly divided into three groups *via* age in the subgroup analysis, including before (20 ≤ age < 45), during (45 ≤ age ≤ 55), and after (age > 55) the perimenopausal period. According to a previous study based on NHANES, circadian variation in sex hormonal factors was adjusted by treating the time of blood sample collection (time of venipuncture) as a covariate, which was classified as morning, afternoon and evening in NHANES (28). However, all participants' blood samples in this study were collected in the morning (see Table 1), so we did not adjust for it. All detailed measurement procedures are publicly available at www.cdc.gov/nchs/nhanes/.

**Table 1:** Baseline characteristics of participants from 2013 to 2016 NHANES, weighted.

**Dietary inflammatory index (DII)**	**Total**	**Tertile 1** **(−4.54 **~**−0.55)**	**Tertile 2** **(−0.55 **~**0.94)**	**Tertile 3** **(0.94 **~**4.28)**	***P*-value**
Participant number	2,092	697	697	698	–
Mean – SD DII	0.21 ± 1.68	−1.66 ± 0.76	0.16 ± 0.43	2.03 ± 0.80	<0.0001
Mean ± SD RIP	2.39 ± 1.63	3.24 ± 1.70	2.90 ± 1.63	2.51 ± 1.58	<0.0001
Mean ± SD Energy intake (kcal)	1,824.05 ± 816.69	2,293.12 ± 858.59	1,814.63 ± 571.88	1,394.02 ± 518.54	<0.0001
Mean ± SD Protein intake (g)	70.36 ± 34.57	91.35 ± 33.98	69.50 ± 26.21	50.10 ± 21.70	<0.0001
Mean ± SD White blood cell count (1,000 cells/uL)	6.95 ± 2.27	6.66 ± 1.93	7.05 ± 2.13	7.13 ± 2.43	<0.0001
Mean ± SD Serum albumin (g/dL)	4.18 ± 0.31	4.25 ± 0.30	4.21 ± 0.30	4.17 ± 0.31	<0.0001
Mean ± SD Total bilirubin (mg/dL)	0.56 ± 0.26	0.58 ± 0.26	0.56 ± 0.26	0.54 ± 0.26	0.0079
Mean ± SD ALT (IU/L)	21.51 ± 15.05	21.19 ± 10.29	22.93 ± 18.50	20.41 ± 13.91	0.0047
Mean ± SD AST (IU/L)	23.72 ± 21.83	23.13 ± 7.90	24.18 ± 14.98	23.98 ± 29.12	0.5465
Mean ± SD TyG index	8.57 ± 0.68	8.47 ± 0.66	8.57 ± 0.66	8.60 ± 0.66	0.0008
Mean ± SD Age (yrs)	49.66 ± 17.30	49.11 ± 16.29	49.95 ± 17.10	48.99 ± 17.73	0.5168
Mean ± SD Total testosterone (ng/dL)	24.73 ± 24.43	25.63 ± 31.90	22.91 ± 14.47	27.83 ± 35.68	0.0059
Mean ± SD Estradiol (pg/ml)	99.02 ± 644.41	114.68 ± 731.52	105.32 ± 711.01	65.78 ± 308.31	0.3090
Mean ± SD SHBG (nmol/L)	74.83 ± 56.20	80.97 ± 59.39	77.08 ± 54.99	76.63 ± 55.35	0.0463
Mean ± SD Bioavailable testosterone (nmol/L)	2.00 ± 3.22	2.03 ± 4.28	1.75 ± 1.75	2.38 ± 4.81	0.0089
**Race (%)**					**0.0001**
Mexican American	16.01	10.25	7.37	7.28	
Other hispanic	12.28	5.62	5.76	6.42	
Non-hispanic white	39.48	67.26	69.55	64.09	
Non-hispanic black	19.31	6.98	10.52	14.87	
Other race	12.91	9.90	6.80	7.35	
**Education level (%)**					**<0.0001**
<9th grade	9.75	4.50	5.00	5.93	
9–11th grade	12.43	7.58	9.71	11.63	
High school graduate	20.60	13.63	21.49	26.25	
Some college or AA degree	30.98	28.91	32.48	39.79	
College graduate or above	26.15	45.25	31.22	16.40	
**Smoking status (%)**					**<0.0001**
Current	16.35	11.59	14.50	23.71	
Former	18.45	23.59	19.09	22.20	
Never	65.11	64.82	66.41	54.09	
**Marital status (%)**					**0.0021**
Married	48.57	60.53	56.21	47.89	
Widowed	10.28	7.83	8.49	9.60	
Divorced	12.33	9.00	11.23	14.66	
Separated	4.02	2.64	2.46	2.62	
Never married	17.11	13.34	15.04	15.65	
Living with partner	7.7	6.66	6.58	9.57	
**BMI (%)**					**0.0007**
Normal (BMI <25 kg/m^2^)	28.49	35.61	14.50	23.71	
Overweight (25 ≤ BMI <30 kg/m^2^)	27.20	18.24	19.09	22.20	
Obese (BMI ≥ 30 kg/m^2^)	43.74	39.27	66.41	54.09	
**Age (%)**					**0.5157**
Before perimenopausal period (20 ≤ age <45)	41.68	42.49	38.73	41.85	
During perimenopausal period (45 ≤ age <55)	16.87	18.24	19.15	16.78	
After perimenopausal period (age ≥ 55)	41.44	39.27	42.12	41.37	
**Time of venipuncture (%)**	–				
Morning	100	100	100	100	

*For continuous variables, P-value was calculated by weighted t-test. For categorical variables, P-value was calculated by the weighted chi-square test. RIP, the ratio of family income to poverty; BMI, body mass index; ALT, alanine aminotransferase; AST, aspartate aminotransferase; TyG index, triglyceride-glucose index*.

### Statistical Analysis

All statistical analyses were performed following CDC guidelines www.cdc.gov/nchs/nhanes/, in which a suitable sample weight was utilized and assigned to each participant in our study (40). Categorical variables are presented as a percentage, and continuous variables are presented as the mean ± standard deviation (S.D.). A weighted chi-square test for categorical variables or ANOVA for continuous variables was employed to analyze differences among different DII tertiles. We performed multivariable linear regression to investigate the association between DII and sex hormones and SHBG. Three different models were assigned to explore the covariate impact on this association (Model 1: no covariates were adjusted; Model 2: age, race, energy intake, and smoking status were adjusted; Model 3: age, race, energy and protein intake, smoking status, RIP, BMI, education level, and marital status were adjusted). The association between BMI and sex hormones was confirmed by accumulative studies (42, 43), so our BMI-stratified subgroup analysis was further conducted using a stratified multivariable linear regression model. The perimenopausal period also had an impact on women's reproductive profiles (25), and we employed another subgroup analysis stratified by the perimenopausal period. Both age and BMI were assigned as prespecified potential effect modifiers. Interaction terms were added to test the heterogeneity of associations between the subgroups (both BMI and perimenopausal period). *P* < 0.05 was considered statistically significant. All analyses were based on R version 4.0.5 (http://www.R-project.org, The R Foundation) and EmpowerStats software (www.empowerstats.com; X&Y solutions, Inc., Boston MA).

## Results

### Weighted Baseline Characteristics of Participants

A total of 2,092 female participants (age ≥ 20) were enrolled in this study after applying the selection and exclusion criteria we mentioned before. We divided baseline characteristics into three continuous DII tertiles (Table 1). The mean ± SD DII was 0.21 ± 1.68, with scores ranging from −4.54 (most anti-inflammatory) to 4.28 (most pro-inflammatory). The three DII tertile ranges were −4.38 to −0.61, −0.60 to 0.93, and 0.93 to 4.21, respectively. The average age and protein and energy intake were 49.66 ± 17.30 years, 70.36 ± 34.57 g, and 1,824.05 ± 816.69 kcal, respectively. Compared with the lowest DII tertile (tertile 1), dramatically decreased protein and energy intakes were observed in the highest (tertile 3), reaching 50.10 ± 21.70 g and 1,394.02 ± 518.54 kcal, respectively. Women in tertile 3 tended to have a higher percentage of non-Hispanic black individuals, obese individuals, and current and former smokers. The mean±SD total testosterone (ng/dL), bioavailable testosterone (nmol/L), estradiol (pg/ml) and SHBG (nmol/L) were 24.73 ± 24.43 ng/dL, 2.00 ± 3.22 nmol/L, 99.02 ± 644.41 pg/ml, and 74.83 ± 56.20 nmol/L, respectively. We found a statistically significant decrease in SHBG levels among the three DII tertiles, with a mean ± SD SHBG of 76.63 ± 55.35 nmol/L in tertile 3 compared with 80.97 ± 59.39 nmol/L in tertile 1. However, total and bioavailable testosterone increased with higher DII scores (P <0.05). A higher DII was also correlated with a higher white blood cell count (tertile 1: 6.66 ± 1.93 vs. tertile 3: 7.13 ± 2.43 1,000 cells/μL, *P* < 0.0001), indicating a more inflammatory condition. Regarding the TyG index reflecting insulin resistance, the DII was positively related to the TyG index (P=0.0008). Total bilirubin and ALT showed a slight decrease in line with a higher DII (*P* < 0.05). Statistically significant differences were also revealed in education level, PIR, and marital status (*P* < 0.05), while age stratified by perimenopausal period and estradiol were not statistically significant (*P* > 0.05).

### The Association Between DII and Sex Hormones and SHBG in the U.S. Females Aged More Than 20 Years

**Figure 2:**
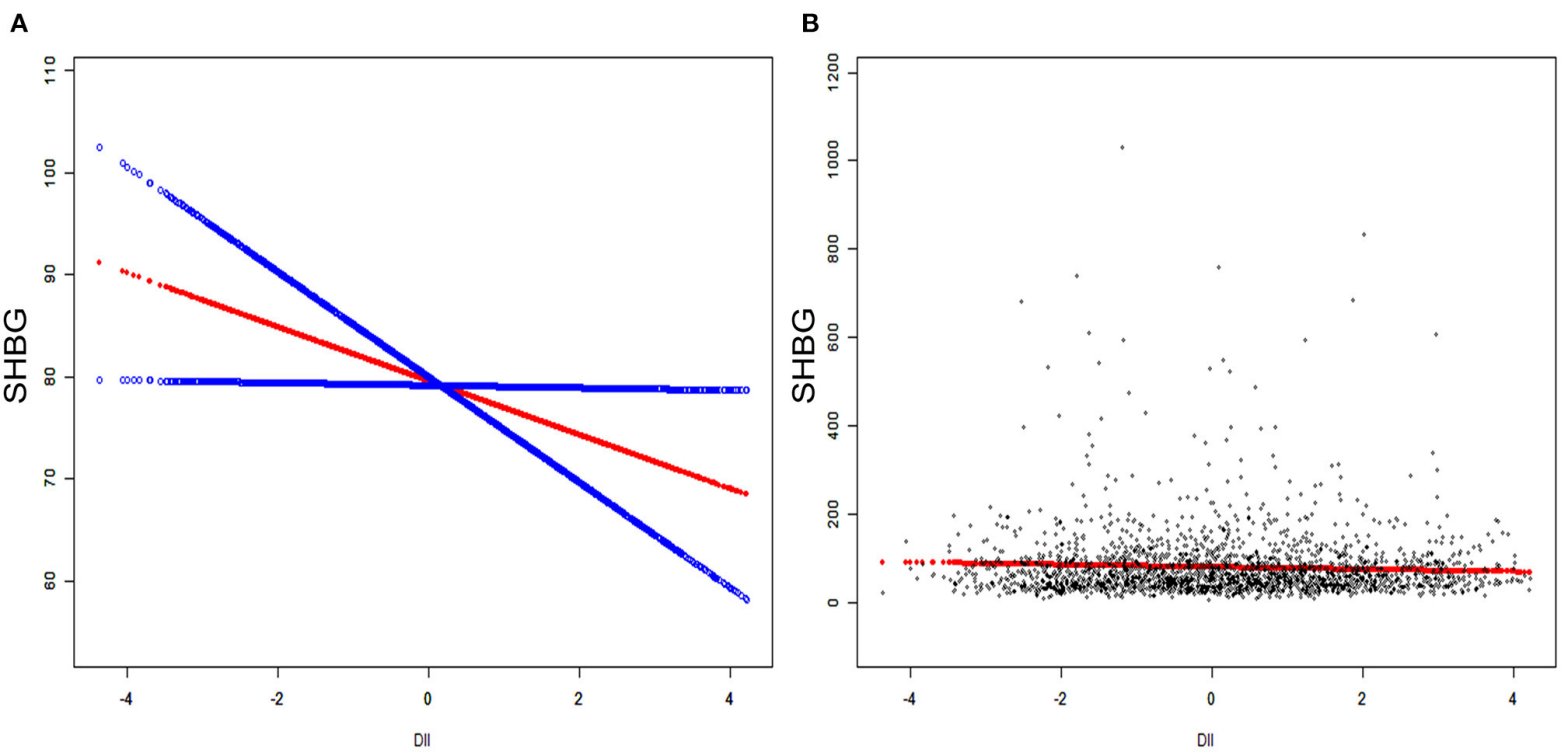
The association between serum DII and SHBG. **(A)** Blue bands represent the 95% CI from the fit. The solid rad line represents the smooth curve fit between variables. **(B)** Each black point represents a single participant SHBG sample.

We applied weighted multivariable regression analysis to evaluate the association among DII, sex hormones and SHBG for females older than 20 years (Table 2). Our results provided a negative but not significant association between DII and SHBG in Models 1 and 2 (Model 1, β = −0.89, 95% CI: −2.38, 0.59, *P* = 0.2394; Model 2, β = −1.41, 95% CI: −3.21, 0.40, *P* = 0.1268). After we adjusted for all covariates, this association became significant (Model 1, β = −2.22, 95% CI: −4.25, −0.18, *P* = 0.0327). There were negative, trace, and non-significant trends among DII and total and bioavailable testosterone (*P* > 0.05). No significant difference was noted in estradiol in any of the three models (*P* =0.3818, 0.5946, 0.1963, respectively). After the DII was divided into tertiles, statistically significant differences became more evident and only existed in SHBG levels. Females in tertile 3 had a much lower SHBG level than those in tertile 1, where the effect size reached −8.05 in the fully adjusted model (Model 3, β = −8.05, 95% CI: −15.59, −0.51, *P* = 0.0366). In the moderately adjusted model (model 2), the effect size referred to as tertile 1 was−5.26 for tertile 2 (Model 2, β = −5.26, 95% CI: −11.37, 0.84, *P* = 0.0913) and −6.25 for tertile 3 (Model 2, β = −6.25, 95% CI: −13.22, 0.73, *P* = 0.0792). The modifier effects of each covariate were summarized in Supplementary Material 1. We utilized smooth curve fittings to characterize the non-linear relationship between DII and SHBG (Figure 2). However, no significant association was found in total and bioavailable testosterone and estradiol levels after DII was divided into groups (all three models, *P* > 0.05).

**Table 2:** The association between dietary inflammatory index and sex hormone & sex hormone binding globulin.

**DII tertiles**	**Model 1^a^**	**Model 2^b^**	**Model 3^c^**
		**β^d^ (95% CI^e^) *P*-value**	
**Total testosterone (ng/dL)**
Continuous	−0.12 (−0.87, 0.63) 0.7538	−0.72 (−1.63, 0.19) 0.1223	−0.82 (−1.90, 0.26) 0.1381
Tertile 1	Ref	Ref	Ref
Tertile 2	−2.72 (−5.67, 0.24) 0.0721	−2.85 (−5.93, 0.22) 0.0694	−3.00 (−6.37, 0.38) 0.0822
Tertile 3	2.20 (−0.84, 5.23) 0.1563	1.08 (−2.44, 4.59) 0.5485	1.54 (−2.46, 5.55) 0.4498
**Bioavailable testosterone (nmol/L)**
Continuous	−0.01 (−0.11, 0.09) 0.8126	−0.09 (−0.21, 0.03) 0.1406	−0.10 (−0.24, 0.05) 0.1954
Tertile 1	Ref	Ref	Ref
Tertile 2	−0.28 (−0.68, 0.11) 0.1584	−0.29 (−0.70, 0.12) 0.1640	−0.34 (−0.79, 0.11) 0.1431
Tertile 3	0.35 (−0.06, 0.75) 0.0941	0.22 (−0.25, 0.69) 0.3692	0.30 (−0.23, 0.84) 0.2669
**SHBG (nmol/L)**
Continuous	−0.89 (−2.38, 0.59) 0.2394	−1.41 (−3.21, 0.40) 0.1268	**−2.22 (−4.25**, **−0.18) 0.0327**
Tertile 1	Ref	Ref	Ref
Tertile 2	−3.89 (−9.75, 1.98) 0.1940	−5.26 (−11.37, 0.84) 0.0913	−4.05 (−10.41, 2.31) 0.2126
Tertile 3	−4.33 (−10.35, 1.69) 0.1586	−6.25 (−13.22, 0.73) 0.0792	**−8.05 (−15.59**, **−0.51) 0.0366**
**Estradiol (pg/ml)**
Continuous	−7.28 (−23.58, 9.03) 0.3818	−5.40 (−25.27, 14.48) 0.5946	−15.00 (−37.76, 7.75) 0.1963
Tertile 1	Ref	Ref	Ref
Tertile 2	−9.36 (−73.76, 55.04) 0.7758	−5.56 (−72.76, 61.63) 0.8711	−16.41 (−87.50, 54.68) 0.6510
Tertile 3	−48.90 (−115.01, 17.22) 0.1473	−49.56 (−126.30, 27.19) 0.2058	−76.05 (−160.35, 8.25) 0.0772

a *Model 1, unadjusted*;

b *Model 2, adjusted for age, race, energy intake, and smoking status*;

c *Model 3, adjusted for age, race, energy & protein intake, smoking status, RIP, white blood cell count, albumin, total bilirubin, ALT, AST, TyG index, marital status, education level, and BMI*.

d *β, regression coefficient*;

e *CI, confidence interval. Statistically significant results were turned bold*.

### Subgroup Analysis

BMI-stratified subgroup analysis was employed and is presented in Table 3. The negative association between DII and SHBG was not statistically significant among the different BMI groups (normal: β = −2.70, 95% CI: −6.56, 1.16, *P* = 0.1703; overweight: β = −2.01, 95% CI: −5.70, 1.69, *P* = 0.2873; obese: β = −1.31, 95% CI: −4.01, 1.38, *P* = 0.3399). Interaction terms (*P* for interaction > 0.05) also confirmed that this negative association was generally stable in different people stratified by BMI groups. After we grouped the DII into tertiles, the results were still not significant among the different BMI groups (P>0.05). However, the results for females from the overweight group were very close to statistical significance (tertile 2: β = −10.69, 95% CI: −21.79, 0.41, *P* = 0.0597; tertile 3: β = −11.64, 95% CI: −25.19, 1.90, *P* = 0.0926). We did not observe significant differences in estradiol in any of the three BMI groups (all *P* > 0.05). The total testosterone level in overweight females was negatively associated with the DII (β = −4.31, 95% CI: −6.80, −1.83, *P* = 0.0007), but this association did not remain significant for bioavailable testosterone (*P* > 0.05). Interestingly, bioavailable testosterone in tertile 3 witnessed a similar increase in both the normal and overweight groups relative to tertile 1. Interaction terms were also added, and no significant difference was found between DII and sex hormones among different BMI groups (for total testosterone, *P* for interaction = 0.1487; for estradiol, *P* for interaction = 0.2759; for bioavailable testosterone, *P* for interaction = 0.6354).

**Table 3:** BMI-stratified subgroup analysis of association between dietary inflammatory index and sex hormone & sex hormone binding globulin.

**DII tertile**	**β**^a^ **(95% CI**^b^**)** P^*^-**value**			
	**SHBG (nmol/L)**	**Estradiol (pg/ml)**	**Total testosterone (ng/dL)**	**Bioavailable testosterone (nmol/L)**
**Normal (<25 kg/m** ^**2**^ **)**
Continuous	−2.70 (−6.56, 1.16) 0.1703	0.94 (−46.81, 48.69) 0.9693	0.62 (−0.55, 1.80) 0.2981	0.09 (−0.06, 0.23) 0.2453
Tertile 1	Ref	Ref	Ref	Ref
Tertile 2	−7.43 (−19.69, 4.83) 0.2353	100.74 (−50.58, 252.06) 0.1925	−2.68 (−6.37, 1.00) 0.1545	−0.16 (−0.62, 0.30) 0.5033
Tertile 3	−6.84 (−21.38, 7.71) 0.3572	−10.14 (−189.62, 169.34) 0.9119	4.32 (−0.05, 8.69) 0.0534	**0.66 (0.11, 1.20) 0.0190**
**Overweight (25–30 kg/m** ^**2**^ **)**
Continuous	−2.01 (−5.70, 1.69) 0.2873	−38.63 (−96.81, 19.56) 0.1938	**−4.31 (−6.80**, **−1.83) 0.0007**	−0.58 (−0.91, −0.24) 0.0008
Tertile 1	Ref	Ref	Ref	Ref
Tertile 2	−10.69 (−21.79, 0.41) 0.0597	−155.22 (−330.22, 19.78) 0.0828	−6.47 (−14.02, 1.08) 0.0937	−0.78 (−1.80, 0.24) 0.1327
Tertile 3	−11.64 (−25.19, 1.90) 0.0926	−224.87 (−438.33, −11.41) 0.0395	−8.15 (−17.36, 1.06) 0.0835	−0.99 (−2.23, 0.25) 0.1180
**Obese (>30 kg/m** ^**2**^ **)**
Continuous	−1.31 (−4.01, 1.38) 0.3399	−4.50 (−22.53, 13.53) 0.6247	0.91 (−1.00, 2.81) 0.3521	0.15 (−0.10, 0.41) 0.2414
Tertile 1	Ref	Ref	Ref	Ref
Tertile 2	0.14 (−8.28, 8.56) 0.9744	−7.66 (−63.99, 48.66) 0.7897	0.29 (−5.65, 6.23) 0.9238	0.07 (−0.72, 0.87) 0.8612
Tertile 3	−3.53 (−13.40, 6.34) 0.4840	−22.29 (−88.34, 43.76) 0.5085	6.90 (−0.07, 13.86) 0.0526	**1.02 (0.08, 1.95) 0.0329**
*P* for interaction	0.1783	0.2759	0.1487	0.6354

a *β, regression coefficient*;

b *CI, confidence interval*;

* *P, P for trend*.

*All presented covariates were adjusted (as Model 3) except BMI categories. Results with statistical significance were turned bold*.

**Figure 3:**
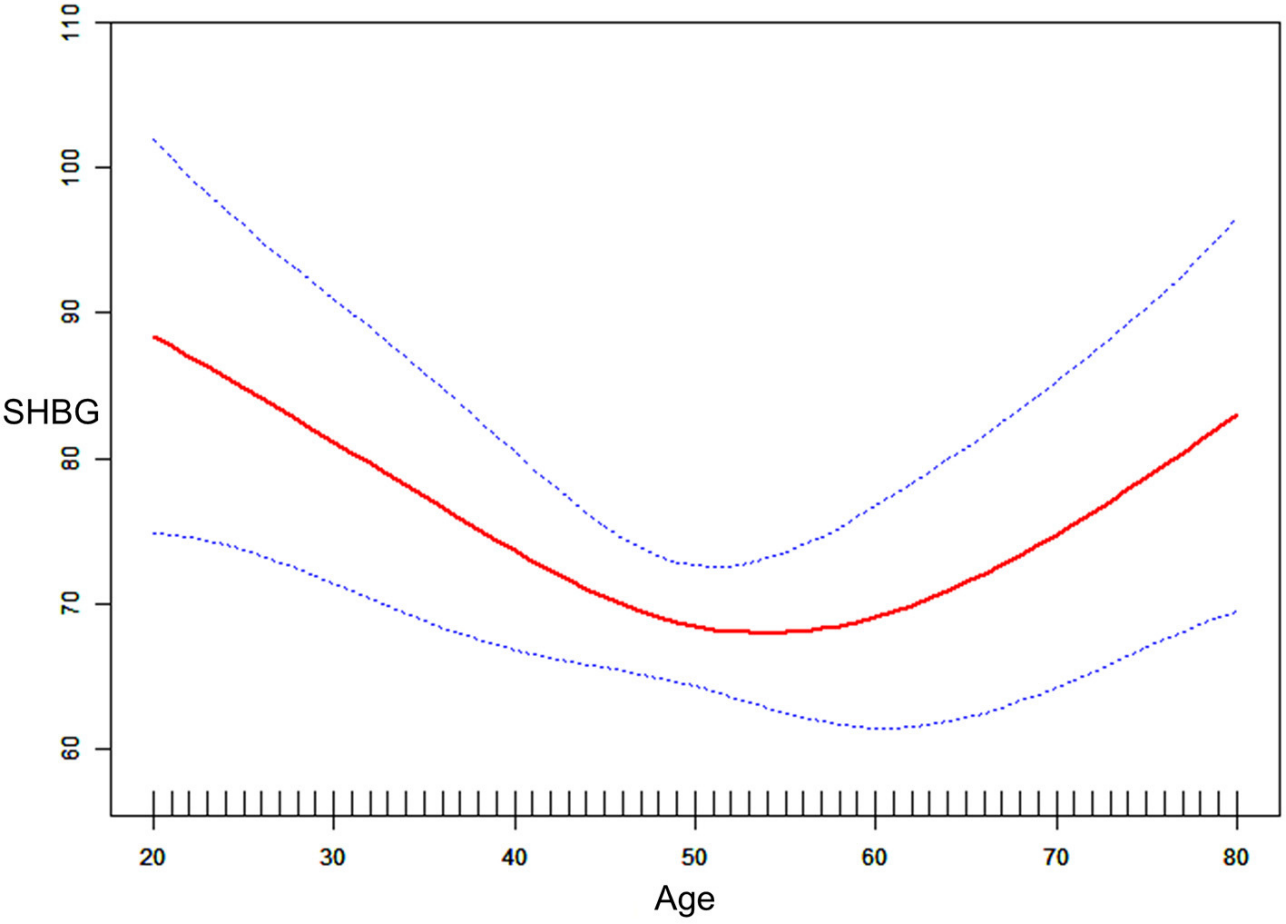
Graphics of smooth curve fittings among age and SHBG.

Perimenopausal period-stratified subgroup analysis was employed and is presented in Table 4. A strong negative relationship between DII and SHBG was observed in ages before (β = −3.71, 95% CI: −7.43, −0.12, *P* = 0.0423) the perimenopausal period. Referring to tertile 1, the effect size in tertile 3 reached −13.39 (95% CI: −27.53, 0.75, *P* = 0.0637) before the perimenopausal period, which was very close to statistical significance. In this period, a slight decrease in estradiol was observed. However, these variations were not statistically significant (*P* > 0.05). For total and bioavailable testosterone in this period, similar declines were identified (total testosterone: β = −2.08, 95% CI: −3.66, −0.49, *P* = 0.0104; bioavailable testosterone: β = −0.24, 95% CI: −0.45, −0.03, *P* = 0.0230). For females during the perimenopausal period, the negative association between DII and SHBG was stable but not significant (*P* > 0.05). We observed increases in both total and bioavailable testosterone for females in tertile 3 (total testosterone: β = 20.27, 95% CI: 4.04, 36.50, *P* = 0.0150; bioavailable testosterone: β = 2.94, 95% CI: 0.70, 5.17, *P* = 0.0105). Although the effect sizes indicated a slight increase in estradiol and SHBG, we did not find statistically significant variation in the after perimenopausal period group (*P* > 0.05). Interactivation terms revealed that there was no significant dependence for the association between DII and SHBG (*P* for interaction = 0.3861), total testosterone (*P* for interaction = 0.3457), bioavailable testosterone (*P* for interaction = 0.4854), and estradiol (*P* for interaction = 0.7256) on different age groups. Specifically, the effect size for SHBG during the perimenopausal period was −0.07, which was reversed from that (0.28) in females after the perimenopausal period. To verify this non-statistically significant association seemingly derived from aging, another smooth curve fittings and generalized additive models were used to characterize the exact relationship between age and serum SHBG, where we converted DII into covariates and age into an exposure variable with serum SHBG remaining as the outcome variable (Figure 3). We identified an inverted *U*-shaped curve in Figure 3 and performed a two-piecewise linear regression model (threshold analysis) to find the exact inflection point (Table 5). This inflection point was age = 50 yrs. For age < 50, a negative association was found between age and SHBG (β = −0.91, 95% CI: −1.46, −0.35, *P* = 0.0014). For age ≥ 50, a per unit increase in age was associated with a 0.77 nmol/L higher level of SHBG (95% CI: 0.24, 1.29, *P* = 0.0041).

**Table 4:** Perimenopausal period–stratified subgroup analysis of association between dietary inflammatory index and sex hormone & sex hormone binding globulin.

**DII tertile**	**β**^a^ **(95% CI**^b^**)** P^*^-value			
	**SHBG (nmol/L)**	**Estradiol (pg/ml)**	**Total testosterone (ng/dL)**	**Bioavailable testosterone (nmol/L)**
**Before perimenopausal period (20** **≤age** **<** **45)**				
Continuous	**−3.71 (−7.43**, **−0.12) 0.0423**	−22.91 (−74.89, 29.06) 0.3878	**−2.08 (−3.66**, **−0.49) 0.0104**	**−0.24 (−0.45**, **−0.03) 0.0230**
Tertile 1	Ref	Ref	Ref	Ref
Tertile 2	−3.43 (−15.56, 8.69) 0.5789	−16.95 (−186.01, 152.10) 0.8442	−3.26 (−8.43, 1.92) 0.2179	−0.42 (−1.11, 0.27) 0.2294
Tertile 3	−13.39 (−27.53, 0.75) 0.0637	−132.55 (−329.70, 64.61) 0.1880	−3.90 (−9.94, 2.14) 0.2059	−0.38 (−1.18, 0.42) 0.3481
**During perimenopausal period (45** **≤age** **<** **55)**				
Continuous	−0.07 (−4.00, 3.86) 0.9725	0.12 (−21.90, 22.14) 0.9916	−0.20 (−4.46, 4.06) 0.9258	−0.02 (−0.61, 0.57) 0.9441
Tertile 1	Ref	Ref	Ref	Ref
Tertile 2	−1.23 (−13.92, 11.46) 0.8496	−34.96 (−105.96, 36.05) 0.3354	−10.60 (−23.93, 2.73) 0.1202	−1.35 (−3.18, 0.49) 0.1518
Tertile 3	−3.84 (−19.29, 11.61) 0.6263	−38.56 (−125.00, 47.87) 0.3826	**20.27 (4.04, 36.50) 0.0150**	**2.94 (0.70, 5.17) 0.0105**
**After perimenopausal period (age** **≥55)**				
Continuous	0.28 (−2.20, 2.75) 0.8274	0.81 (−0.60, 2.23) 0.2606	**1.19 (0.29, 2.09) 0.0095**	**0.13 (0.03, 0.23) 0.0149**
Tertile 1	Ref	Ref	Ref	Ref
Tertile 2	−6.45 (−13.75, 0.86) 0.0842	2.73 (−1.46, 6.93) 0.2018	0.80 (−1.87, 3.47) 0.5571	0.19 (−0.11, 0.49) 0.2183
Tertile 3	−1.89 (−10.56, 6.78) 0.6691	4.42 (−0.55, 9.40) 0.0817	2.77 (−0.39, 5.94) 0.0861	0.32 (−0.04, 0.68) 0.0843
*P* for interaction	0.3861	0.7256	0.3457	0.4854

a *β, regression coefficient*;

b *CI, confidence interval*;

* *P, P for trend*.

*All presented covariates were adjusted (as Model 3) except age categories. Results with statistical significance were turned bold*.

**Table 5:** Threshold effect analysis of age on SHBG level in adult females using the two-piecewise linear regression model.

**SHBG (nmol/L)**	**β (95% CI) *P*-value**
Standard linear model	−0.02 (−0.40, 0.36) 0.9189
**Two-piecewise linear model**
Inflection point	50
20 ≤ Age <50 (yrs)	−0.91 (−1.46, −0.35) 0.0014
Age ≥ 50 (yrs)	0.77 (0.24, 1.29) 0.0041
Log likelihood ratio	<0.001
Effect size deviation	1.67 (0.91, 2.43) <0.0001

*Age was converted into exposure variable. age, race, energy & protein intake, smoking status, RIP, white blood cell count, albumin, total bilirubin, ALT, AST, TyG index, marital status, education level, and BMI were adjusted*.

## Discussion

Two thousand and ninety-two female participants aged more than 20 years were enrolled in this cross-sectional study, where we revealed that higher consumption of a proinflammatory diet (higher DII) for women was negatively associated with serum SHBG levels after we adjusted for all potential covariates. No statistically significant association was revealed between DII and estradiol and total and bioavailable testosterone. In BMI-stratified subgroup analysis, the negative associations between DII and SHBG were stable but not statistically significant across different BMI groups. We did not observe a significant dependence on BMI itself on these associations *via* interaction terms. In perimenopausal period-stratified subgroup analysis, we identified a negative association between DII and SHBG in women before the perimenopausal period.

Since sex hormones and SHBG are easily affected by inflammation, we utilized the DII, an important literature-derived dietary tool to assess dietary inflammatory potential, to investigate the impact of dietary patterns on sex hormonal factors in the NHANES population. Unlike similar NHANES cross-sectional study in males, the relationships between DII and sex hormone (28, 29), including bioavailable and total testosterone, and estradiol were weak and unstable in general U.S. adult females. We found a negative relationship between DII and SHBG in U.S. adult females after we adjusted for all potential covariates in our study, which might account for the U.S. national prevalence of low SHBG. It is well-established that low SHBG could lead to multiple unfavorable health outcomes, including a higher risk of PCOS, breast cancer incidence and mortality, type 2 diabetes (T2D), and several cardiovascular diseases (7–10). To prevent these unfavorable health outcomes, our study addressed that managing dietary patterns could be a potential method for sex hormonal factor-related disease prevention in women's health throughout their lives.

To our knowledge, this is the first study assessing the association between the overall dietary inflammatory potential and sex hormones and SHBG in adult women. Probable diet-induced reproductive profile changes have become a hot topic in recent years. Early studies found that ingestion of flax seed powder (rich in lignans) could play a role in ensuring a normal ovulation cycle with a longer luteal phase (L.P.) lengths and higher L.P. progesterone/estradiol ratio (44). A cohort study containing 270 Caucasian women delivered in a Boston hospital revealed that SHBG was positively associated with vegetable and pulse intake. In contrast, pregnancy progesterone was positively associated with alcohol intake and inversely associated with polyunsaturated lipid and vitamin B12 intake (45). Karelis et al. found that vegetarians presented higher concentrations of SHBG in both pre- and post-menopausal women due to higher levels of fiber intake (46). Two dietary patterns, known as intermittent continuous energy (IER) and continuous energy restriction (CER), were found to both be useful to improve SHBG levels in young overweight women (47). For women with PCOS, where abnormally low serum SHBG levels are frequently found, therapeutic interventions improved SHBG levels in PCOS women, which further reduced PCOS-associated complications (7). In fact, dietary management of SHBG levels was thought to be effective in PCOS patients. The ketogenic Mediterranean diet was also considered a useful non-pharmacological treatment for PCOS patients, leading to a decrease in the Ferriman Gallwey Score and increases in estradiol, progesterone, and SHBG (48). The hypocaloric low glycemic index (LGI) diet contributed to healthy weight loss, improved SHBG, and decreased homeostatic model assessment of insulin resistance (HOMA-IR) in people with and without PCOS. Mehri Jamilian et al. found that high-dose vitamin D supplementation for 12 weeks could have beneficial effects on total testosterone, SHBG, free androgen index, and serum CRP levels compared with low-dose vitamin D and placebo groups for insulin-resistant patients with PCOS (49). Based on our results, a proinflammatory diet could lead to a SHBG decrease, which might aggravate PCOS-related symptoms.

Since the population age of this study is more than 20, it includes the female reproductive period and perimenopausal and postmenopausal periods. Among them, perimenopause is initiated with irregular menstrual cycle lengths, and follicle-stimulating hormone (FSH) concentrations rise in response to decreased concentrations of ovarian hormones (39). A strong negative association between DII and SHBG was observed only in women before and during the perimenopausal period from our subgroup analysis stratified by the perimenopausal period. This association did not remain statistically significant for women after the perimenopausal period. Moreover, a negative association between DII and total testosterone was only found in women during the perimenopausal period. Since perimenopause could induce reproductive profile disorder, we thought this disorder might account for this association among the “during the perimenopausal period” subgroup. After we transformed the DII into covariates, smooth curve fittings and threshold analysis identified a non-linear relationship between age and SHBG, with a point of inflection at 60 yrs. These findings correlated with a previous study, which showed that SHBG concentration was naturally higher in postmenopausal women than in women in other stages (50). Laughlin et al. also reported that SHBG levels increased approximately 30% in women between 50 and 89 years old, probably accounting for the bound testosterone concentration increases (51).

In addition, two recent studies obtaining data from NHANES confirmed that a higher DII was associated with decreased total testosterone and estradiol levels in male adolescents and decreased total testosterone levels in male adults (28, 29). However, our study found that a higher DII might mainly contribute to SHBG decrease and a comparatively slight total testosterone decrease in adult females. The sex difference occurred among these studies, and we suspected that women-specific characteristics, such as menstrual cycle, perimenopause, pregnancy, and delivery, could all contribute to this condition. Previous dietary intervention studies in children demonstrated that a low cholesterol diet for male children did not affect serum hormone levels, SHBG, or Tanner stages, whose results were almost completely different from those of a similar study conducted on girls (52, 53). However, a diet with higher inflammatory potential poses undesirable impacts on reproductive profiles.

Although the exact mechanism for our results remains unclear, emerging evidence indicates that a proinflammatory diet-induced SHBG decline might rely on increased proinflammatory cytokines, such as IL-1, IL-6, IL-17, and tumor necrosis factor (TNF). TNF-α rather than insulin could be the main factor accounting for decreased SHBG levels *via* nuclear factor kappa B (NF-κB) and then downregulating HNF-4α mRNA in human hepatoblastoma-derived (HepG2) cells (54). This study also observed decreased SHBG levels through NF-κB in human SHBG transgenic mice. Consistently, SHBG levels rebounded in patients with chronic inflammatory states, such as rheumatoid arthritis, using a TNF-α inhibitor (55). High plasma IL1β levels reduced SHBG synthesis in HepG2 cells by inhibiting HNF-4A *via* the MAPK kinase (MEK)-1/2 and c-Jun N-terminal kinase (JNK) MAPK signaling pathways through the activation of c-Jun transcription factors (56). Daily IL1β supplementation in human SHBG transgenic mice reduced plasma SHBG and SHBG mRNA levels (56). The concentration of SHBG and the levels of SHBG mRNA were dose-dependently decreased in the presence of IL-6 concentrations ranging from 0.1 to 10 ng/mL, where dexamethasone had a significant additive effect on IL-6 inhibition of SHBG secretion and mRNAs in HepG2 cells (57). Since carbohydrates were assigned as a proinflammatory food parameter in the DII calculation, the downregulation of SHBG could derive from excessive carbohydrate consumption. hSHBG transgenic mice fed high amounts of sucrose or glucose exhibited decreased human serum SHBG levels, whereas *in vitro* studies revealed that excessive monosaccharides directly downregulated SHBG gene expression by increasing *de novo* lipogenesis (58). Palmitate treatment could also reduce SHBG production by reducing HNF-4A levels in HepG2 cells (58).

This study has several strengths. This is the first study to examine the association between DII and sex hormones and SHBG among adult females in a nationally representative U.S. population. This study adjusted for potential confounders in batches via three different models. The exposure variable (DII) was treated as a simple continuous variable and categorized into tertiles. Subgroup analyses stratified by BMI or perimenopausal period were carefully established, followed by interaction terms to test heterogenicity among different subgroups.

However, our study also has several limitations. First, this is a cross-sectional study based on NHANES, so we cannot disentangle the exact temporal relationship. A subsequent large-scale cohort study is necessary to further confirm our results. Second, the DII calculation was based on 24-h dietary recall; RIP and martial & smoking status were obtained from questionnaires in NHANES, where recall bias is inevitable. Third, serum sex hormone and SHBG concentrations were detected only at a single time point, and the specific timing was not recorded in NHANES, which cannot represent their diurnal changes. Although we adjusted the time of venipuncture, this bias could be inevitable. Fourth, diagnoses of hypogonadism and fatty liver, which could change sex steroid & SHBG production, were absent in NHANES. Last, distinction between clinical symptoms related specifically to the menopausal transition and those related to aging in general was difficult. Our definition of the perimenopausal period in the subgroup analysis was incomplete.

## Capsule

Dietary inflammatory potential was negatively associated with SHBG in U.S. adult females.

## Data Availability Statement

The original contributions presented in the study are included in the article/Supplementary Material, further inquiries can be directed to the corresponding author/s.

## Author Contributions

NL and YF contributed to the conception, software, original draft writing and design, acquisition, analyses, and interpretation of data. XL contributed to the methodology, formal analyses, and writing—review and editing. XM and FM revised this work, provided funding, and gave some important suggestions. All authors contributed to the article and approved the submitted version.

## Funding

This work was supported by the National Natural Science Foundation of China (31771662) and the National Key Research and Developmental Program of China (2018YFC1002803).

## Conflict of Interest

The authors declare that the research was conducted in the absence of any commercial or financial relationships that could be construed as a potential conflict of interest.

## Publisher's Note

All claims expressed in this article are solely those of the authors and do not necessarily represent those of their affiliated organizations, or those of the publisher, the editors and the reviewers. Any product that may be evaluated in this article, or claim that may be made by its manufacturer, is not guaranteed or endorsed by the publisher.
