# Supplementary material for: Association Between Dietary Inflammatory Index and Sex Hormone Binding Globulin and Sex Hormone in U.S. Adult Females
Source: Front Public Health. 2022 Apr 15;10:802945. doi: 10.3389/fpubh.2022.802945 (PMC9051085; doi:10.3389/fpubh.2022.802945)
Supplement: Supplementary file 1 [file Table_1.DOCX]

**Supplementary Material 1. The modifier effects of each covariate in the association between DII and sex hormonal factors.**

| Variable | SHBG | Total testosterone | Bioavailable testosterone | Estradiol |
| --- | --- | --- | --- | --- |
|  | β, SE, P-value | β, SE, P-value | β, SE, P-value | β, SE, P-value |
| DII | -2.22, 0.91, 0.0327 | -0.82, 0.42, 0.1381 | -0.10, 0.55, 0.1954 | -15.00, 11.03, 0.1963 |
| RIP | 1.62, 0.87, 0.0654 | -0.52, 0.41, 0.2105 | -0.06, 0.05, 0.2191 | -7.42, 10.74, 0.4892 |
| Serum albumin | -45.94, 4.26, <0.0001 | -2.39, 2.01, 0.2340 | 0.29, 0.26, 0.2646 | -354.28, 52.17, <0.0001 |
| TyG index | -3.99, 2.00, 0.0463 | -3.71, 0.94, <0.0001 | -0.35, 0.12, 0.0044 | 14.71, 24.51, 0.5485 |
| White blood cell count | -1.49, 0.61, 0.0152 | 0.51, 0.29, 0.0754 | 0.10, 0.04, 0.0066 | 24.12, 7.49, 0.0013 |
| AST | 0.16, 0.08, 0.0396 | -0.03, 0.04, 0.4022 | -7.80*10^-3^, 4.71*10^-3^, 0.0979 | -0.23, 0.94, 0.8038 |
| ALT | -0.24, 0.11, 0.0385 | 0.14, 0.05, 0.0105 | 0.02, 7.02*10^-3^, 0.0007 | -0.76, 1.40, 0.5827 |
| Total bilirubin | -6.77, 4.79, 0.1579 | 3.04, 2.26, 0.1776 | 0.55, 0.29, 0.0607 | -7.08, 58.64, 0.9038 |
| Energy intake | 0.01,0.01, 0.9544 | -0.01, 0.001, 0.8099 | -1.21*10^-5^, 1.38*10^-4^, 0.2191 | 0.02, 0.03, 0.3786 |
| Protein intake | 0.03, 0.05, 0.6093 | 0.01, 0.03, 0.9800 | 2.86*10^-4^, 3.41*10^-3^, 0.9331 | -0.59, 0.68, 0.3797 |
| Race | | | | |
| Mexican American | Ref | Ref | Ref | Ref |
| Other Hispanic | -5.93, 4.63, 0.2008 | 1.90, 2.19, 0.3836 | 0.26, 0.28, 0.3578 | -125.92, 56.80, 0.0267 |
| Non-Hispanic White | 8.46, 3.86, 0.0287 | 0.13, 1.82, 0.9439 | -0.16, 0.24, 0.4983 | -78.57, 47.33, 0.0971 |
| Non-Hispanic Black | 1.60, 4.42, 0.7181 | 0.03, 2.09, 0.9888 | -0.07, 0.27, 0.7841 | -84.39, 54.18, 0.1195 |
| Other Race | -12.75, 4.82, 0.0083 | -1.06, 2.28, 0.6421 | 0.04, 0.30, 0.8868 | -114.59, 59.06, 0.0525 |
| Smoking status | | | | |
| Current | Ref | Ref | Ref | Ref |
| Former | 1.23, 4.12, 0.7650 | -2.67, 1.95, 0.1695 | -0.33, 0.25, 0.1954 | 11.01, 50.48, 0.8273 |
| Never | 0.75, 3.49, 0.8295 | -5.43, 1.65, <0.0001 | -0.62, 0.21, 0.0039 | 46.79, 42.72, 0.2736 |
| Education level | | | | |
| Less than 9th grade | Ref | Ref | Ref | Ref |
| 9-11th grade | -4.49, 5.36, 0.4021 | 1.91, 2.19, 0.3836 | 0.43, 0.33, 0.1873 | 1.35, 65.65, 0.9836 |
| High school graduate | -6.06, 5.00,0.2258 | 0.13, 1.82, 0.9439 | 0.77, 0.30, 0.0118 | 21.89, 61.28, 0.7210 |
| Some college or AA degree | -2.99, 4.83, 0.5357 | 0.03, 2.08, 0.9889 | 0.52, 0.29, 0.0782 | 15.73, 59.19, 0.7904 |
| College graduate or above | 1.71, 5.28, 0.7465 | -1.06, 2.28, 0.6421 | 0.51, 0.32, 0.1171 | 73.02, 64.70, 0.2592 |
| Marital status | | | | |
| Married | Ref | Ref | Ref | Ref |
| Widowed | 5.83, 4.19, 0.1646 | -1.72, 1.98, 0.3836 | -0.26, 0.26, 0.3136 | -133.84, 51.40, 0.0093 |
| Divorced | 3.44, 3.84, 0.3707 | 0.40, 1.82, 0.8254 | 0.07, 0.24, 0.7617 | -51.86, 47.10, 0.2710 |
| Separated | 11.70, 6.32, 0.0646 | -0.34, 2.98, 0.9085 | -0.41, 0.39, 0.2892 | 2.96, 77.49, 0.9695 |
| Never married | 0.26, 3.50, 0.9418 | 2.46, 1.65, 0.1377 | 0.28, 0.22, 0.1875 | -26.44, 42.96, 0.5383 |
| Living with partner | 11.60, 4.63, 0.0124 | 1.62, 2.19, 0.4592 | 0.05, 0.28, 0.8528 | 15.48, 56.76, 0.7851 |
| BMI | | | | |
| Normal | 47.10, 3.32, <0.0001 | -0.34, 1.57, 0.8269 | -0.70, 0.20, 0.0006 | 128.69, 40.71, 0.0016 |
| Overweight | 20.08, 2.97, <0.0001 | 0.69, 1.41, 0.6253 | -0.21, 0.18, 0.2606 | 114.17, 36.45, 0.0018 |
| Obese | Ref | Ref | Ref | Ref |
| Age | | | | |
| 20≤age<45 | -3.33, 2.73, 0.2215 | -1.28, 1.29, 0.3192 | -0.13, 0.17, 0.4545 | -113.59, 33.41, 0.0007 |
| 45≤age<55 | -9.13, 3.45, 0.0081 | -3.67, 1.62, 0.0243 | -0.34, 0.21, 0.1093 | -102.49, 42.21, 0.0152 |
| age≥55 | Ref | Ref | Ref | Ref |

β, regression coefficient; SE, standard error; DII, dietary inflammatory index; RIP, the ratio of family income to poverty; TyG index, triglyceride-glucose index; ALT, alanine aminotransferase; AST, aspartate aminotransferase; BMI, body mass index.
